# Eye care for older people

**Published:** 2008-06

**Affiliations:** Lecturer, International Centre for Eye Health, London School of Hygiene and Tropical Medicine, Keppel Street, London WC1E 7HT, UK. Email: jennifer.evans@Lshtm.ac.uk

**Figure:**
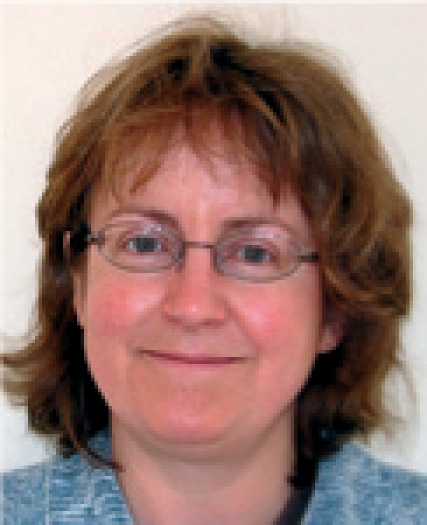


## An ageing population: the global trend

Worldwide, people are now living longer and birth rates are declining; older people are therefore making up an ever greater proportion of the world's population. This means that the number of older people is increasing very rapidly compared to the overall growth of the global population.

By 2025, there will be twice as many older people worldwide as there were in 2000 (an increase from 606 million to 1.2 billion). Twenty-five years later, by 2050, the population of older people will be three times greater than in 2000: around two billion.^1^

This trend will be experienced, to varying degrees, in countries all over the world. In Africa, the number of people aged 60 and above is expected to increase at double the worldwide rate; it is estimated that this population will grow from 38 million in 2000 to at least 203 million in 2050. In fact, it is estimated that 75 per cent of older people will be living in low- and middle-income countries by 2025.

**Figure:**
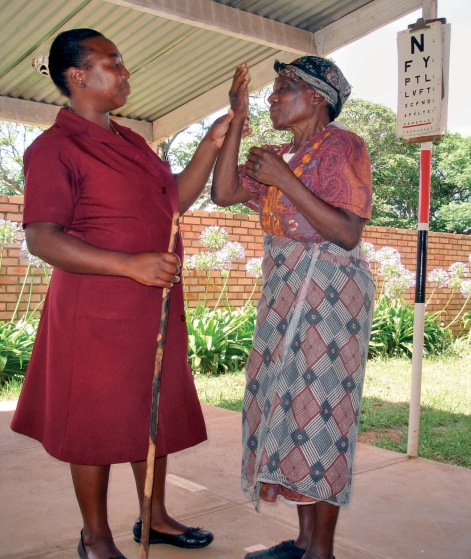
**An older women has her vision tested. SWAZILAND.**

## Impact of ageing on eye care systems

For many low-income countries, such an increase in the number of older people will be particularly challenging as this change in the population may take place before there has been sufficient economic development to deal with its effects. In particular, an ageing population will place an extra burden on the health care system in general.

The prevalence of visual impairment increases with age. Although people aged 50 years and above only represent 19 per cent of the world's population, more than 82 per cent of people living with blindness are in this age group.^2^ A rise in the number of older people in a population will therefore be accompanied by an increase in the number of people with age-related eye diseases, such as cataract and age-related macular degeneration.

The costs associated with treatment and rehabilitation can be expected to increase dramatically over the next few decades.

In lower-income countries, different generations tend to live under one roof and the role of caring for older people falls mainly to the family. Relatives also have to meet the costs associated with treatment and rehabilitation.

In many middle- and high-income countries, however, older people are becoming increasingly isolated. This is due to the breakdown of the family unit and the need for working-age relatives to move in order to find employment. As people age alone, without family members to look after them, governments might face increased pressure to provide care; this could include meeting the costs of treatment and rehabilitation.

## Impact on society

Not all the costs associated with an increase in age-related diseases are financial. Visual impairment has a negative impact on the lives of older people, their families, and society as a whole.

Older people with good vision can, and do, remain economically and socially active as they age, and they contribute significantly to the wellbeing of their families and to society in general (see article on page 24). This contribution is particularly important in populations affected by HIV and AIDS, where children may have lost both their parents and are looked after by grandparents. Unfortunately, visual impairment dramatically reduces the ability of older people to contribute to their full capacity, which has a negative impact on society as a whole.

## What can be done about the impact of ageing on eye health?

Considerable resources are needed to help older people overcome the limitations imposed by poor vision.

### Improving access to treatment

Despite the fact that much visual impairment in older people is due to correctable conditions such as refractive error and cataract, older people in many countries still suffer from these conditions. Even in high-income countries such as the UK, where good quality eye care is free at the point of delivery, there are high levels of visual impairment in older people.^3^ This problem is particularly acute among older people who do not live in the community, for example, people living in residential homes or nursing homes.

Older people face particular challenges when accessing health care, including eye care. One of the reasons is that, as people age, many health problems can occur at the same time. In the presence of multiple health problems, vision problems probably assume a lesser importance; in addition, these other health problems can make it physically more difficult for an older person to access eye care. Expectations and activities also decrease with age, which affects older people's desire to seek help with their health problems, including eye problems.

The article on page 26 discusses these and other challenges older people face when trying to access eye care and offers some suggestions for overcoming them.

### Prevention

Although safe and effective treatment for cataract is available, the costs to society of dealing with this problem on such a wide scale may become very high. In addition, the new treatments emerging for age-related macular degeneration are currently too costly to benefit more than a small portion of those suffering from this condition.

Prevention of cataract and age-related macular degeneration may therefore become particularly important in the future. Although we hope to uncover more evidence about the prevention of these conditions, there already exists very clear evidence that tobacco smoking is a risk factor for both conditions.

Almost one billion men in the world smoke, but levels of smoking in men appear to be declining.^4^ Although the rates of smoking in women are declining in high-income countries, this is by no means the case worldwide. Overall, healthier, better educated people are heeding public health warnings on the dangers of smoking. However, smoking is now becoming much more concentrated in poorer, less educated populations; this is precisely the group which has been shown to be in worse health and to have more limited access to health care.

Therefore, there should be a focus on public health interventions in poorer, less educated populations to reduce levels of smoking.

The relationship between nutrition during life and age-related eye diseases is currently being investigated. Firm public health recommendations on this issue can not be made at present. It is more than likely, however, that a healthy and active old age requires adequate levels of nutrition at all stages in life.

Maintaining good vision is an important part of ‘active ageing’, a concept promoted by the World Health Organization. Active ageing means: continued health, security, and participation in society as people age, in order to ensure a good quality of life in later years.^5^ As eye care practitioners, therefore, we should work together with other health and social services to help those in our care remain as active as possible in their later years.

## Changing attitudes to ageing

Changes in attitudes towards ageing and older people, which differ in different parts of the world, will no doubt influence the consequences of ageing in different regions.

On the one hand, societies traditionally respectful towards older people are finding that the gradual globalisation of culture can lead to a ‘culture of youth’. This can mean that youth is idealised and that there is less respect towards older people.

On the other hand, because the number of older people is increasing as a proportion of the total population, their voices may have a better chance of being heard. This is particularly true in high-income countries, where this new phenomenon has been called ‘grey power’ or ‘grey dollar’ to indicate that the commerce and industry sector is beginning to appreciate the economic power of these older consumers. This societal change is reinforced by the fact that, in these countries, the new generations of older people are used to being vocal and politically active. This may result in increased respect for, and responsiveness to, the needs of older people.

With increasing health awareness in all countries, we may also hope for a ‘generational effect’: it is possible that current generations, more used to being ‘consumers’ of health care, will engage actively with health care services in their later years. Rapid changes in access to information, thanks to the internet and other digital media, will undoubtedly have an effect as users of health care services will become more informed about what is available to them.

## Conclusion

For any country, an ageing population is something to be proud of; it is a success story reflecting the fact that people are living longer and healthier lives. However, it is important to plan for the effects of an ageing population, both in terms of health care provision and public health campaigns. Appropriate public health messages should be provided at all ages to encourage healthy lifestyles that promote eye health in the long term. We must also ensure that older people have good access to eye care services.

**When is someone an older person?**

Most high-income countries use the chronological age of 65 years to define older people. For Africa, the current recommended cutoff is 50 years. Although the United Nations currently have no standard numerical criterion, the generally accepted cutoff is 60 years and above. (www.who.int)

**Summary**

- Populations are ageing because of increased life expectancy and decreased birth rates.
- Most high-income countries already have large populations of older people. Low- and middle-income countries will experience a rapid increase in the number of older people in their populations.
- With increasing age, the prevalence of visual impairment and age-related diseases, such as cataract and age-related macular degeneration, increases rapidly. This has implications for eye health and social care costs.
- Public health interventions should focus on reducing the levels of smoking in the population to reduce the incidence of age-related cataract and age-related macular degeneration.
- Older people have different needs, which should be taken into consideration by eye care providers. They also face more barriers to health care, older women in particular.
- Good vision can help older people remain active for longer.

**World Sight Day 2008: Eyes on the Future**

**Figure:**
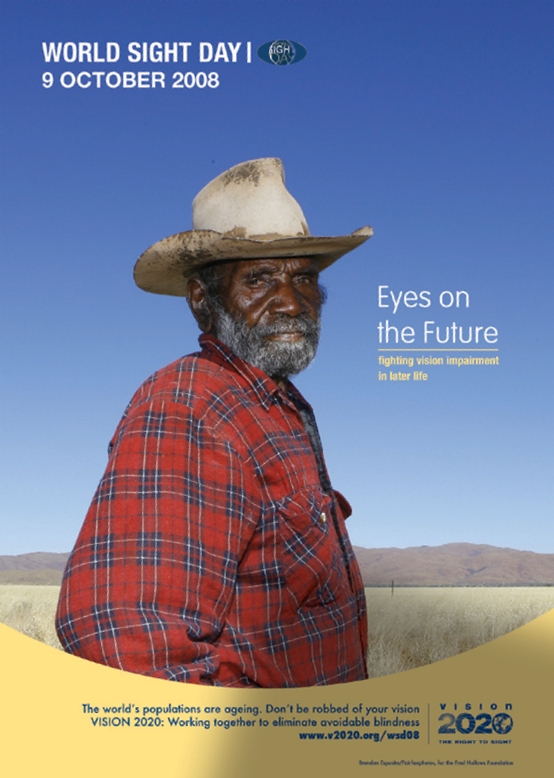
Photo courtesy of Brendan Esposito/Fairfaxphotos and The Fred Hollows Foundation

**Figure:**
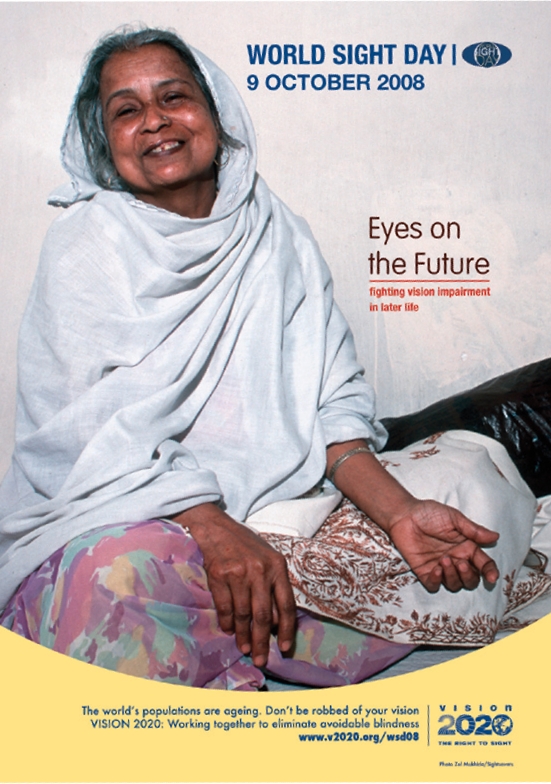
Photoi Zul Mukhida/Sightsavers

Did you know that 80 per cent of the world's 45 million blind people are over 50 years of age?

The theme of World Sight Day 2008 is the ageing eye and visual impairment in older people. The headline “Eyes on the Future” recognises that, in a world where populations are ageing and individuals are living longer, blindness from chronic conditions is also on the increase.

World Sight Day is the annual day of awareness for blindness and visual impairment and is held on the second Thursday of October (this year it is on 9 October 2008). Included on the official calendar of the World Health Organization, it is coordinated by IAPB under the VISION 2020 Global Initiative. The global theme and certain promotional materials are generated by IAPB.

All World Sight Day events are organised independently by members and supporters of VISION 2020. IAPB encourages all organisations concerned with eye health to arrange events and displays to mark this special day. Ideas for events include competitions, eye screening camps, sponsored walks, and gala dinners. Promotional posters, bookmarks, and official literature can be requested from IAPB. See www.v2020.org/wsd08 for more details.
